# Adjuvant Radiotherapy for Synchronous Bilateral Testicular Seminoma: A Case Report and a Review of the Pertinent Literature

**DOI:** 10.1155/2013/241073

**Published:** 2013-05-28

**Authors:** Daniel A. Jones, Elizabeth C. Ester, David Leavitt, Robert Sweet, Badrinath Konety, Gautam Jha, L. Chinsoo Cho

**Affiliations:** ^1^Department of Radiation Oncology, University of Minnesota Medical Center, Minneapolis, MN 55455, USA; ^2^Department of Urology, University of Minnesota Medical Center, Minneapolis, MN 55455, USA; ^3^Department of Medicine, Division of Hematology, Oncology, Bone Marrow Transplantation, Masonic Cancer Center, University of Minnesota Medical Center, Minneapolis, MN 55455, USA

## Abstract

Few cases of synchronous bilateral stage I seminomas have been reported in the world literature. We present a case of bilateral synchronous testicular seminoma, the current literature on the management of stage I seminoma, and the implications for radiotherapy. A forty-year-old man presented with synchronous bilateral classical seminomas, both stage IA. After undergoing bilateral inguinal orchiectomy, he received adjuvant external beam radiotherapy, with a standard paraaortic field. After 18 months of followup, he remains well, without evidence of recurrence. Bilateral germ cell tumors (BGCTs) are reported consistently at a low rate. Bilateral radical inguinal orchiectomy is standard of care, yet some groups have proposed an organ preservation approach. Of the reported cases of bilateral stage I synchronous GCT, with concordant seminoma histology, most of them were treated with bilateral orchiectomy and adjuvant radiotherapy. Although morbidity associated with radiotherapy directed at the abdomen is not negligible, adjuvant paraaortic radiotherapy remains safe and well-tolerated treatment regime. Bilateral synchronous stage I seminoma of the testes is rare. Organ preservation remains investigational. Chemotherapy is probably a reasonable option. We propose that patients with bilateral stage I synchronous GCT, with concordant seminoma histology, should be managed with bilateral orchiectomy, followed by paraaortic radiotherapy.

## 1. Introduction

According to a publication by the American Cancer Society, there was an estimated 8590 new cases of testicular cancer in the United States in 2012, accounting for only 360 deaths [1]. Testicular germ cell tumors (GCTs) make up a majority of these cases. Men diagnosed with testicular GCT are at higher risk for development of a second cancer in the contralateral testes, and the incidence of bilateral testicular germ cell tumor (BGCT) ranges from 1% to 4% in selected series, as highlighted in Table 1 [2–12].

Furthermore, the incidence of synchronous BCGT is much less common, accounting for less than 0.5% of all diagnoses of testicular cancer. Of the synchronous presentations of BGCT, it is quite rare to see a presentation of stage I synchronous concordant seminoma in both testes, as indicated in Table 2 [2–4, 6, 7, 9–12]. Most of these patients have been managed initially with bilateral orchiectomy, followed by adjuvant radiation therapy. Field sizes, dose, and fractionation regiments generally were not reported. Nearly all patients, in this small cohort, at the time of publication had no evidence of disease NED (Table 2). By our review of the literature, there have been no reported cases of observation after bilateral orchiectomy of synchronous seminomatous BGCT. As the paradigm of management for stage I seminoma is shifting to a more conservative approach, for bilateral testicular germ cell tumors, adjuvant radiotherapy or chemotherapy probably remains the best option.

## 2. Case Presentation

**Figure 1:**
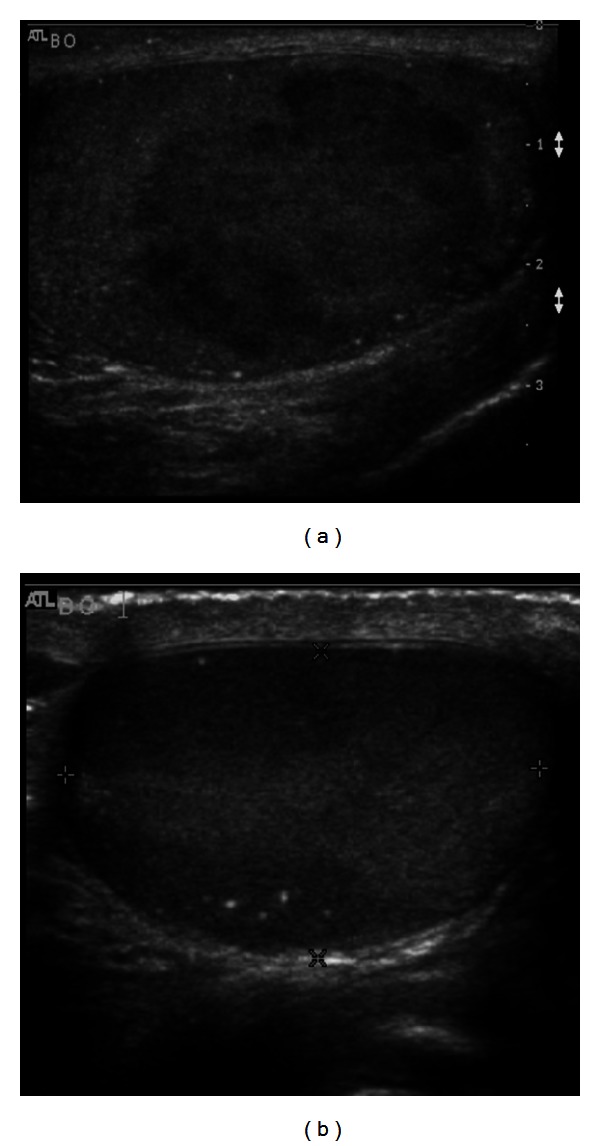
Scrotal ultrasound. (a) (R), (b) (L).

The patient is a forty-one-year-old man with an unremarkable medical history presented with a two-month history of scrotal swelling and discomfort. He denied a history of mal-descent, and there was no family history of testicular cancer. Physical exam was pertinent for an enlarged, nontender left testicle. An ultrasound revealed well-circumscribed hypo-echoic, heterogeneous lesions in both testicles (Figure 1). The left testicular mass measured 3.0 × 2.6 × 4.3 cm, while the mass in the right testicle measured 2.1 × 3.1 × 0.5 cm. Doppler was suggestive of normal blood flow. Pertinent labs included (AFP) alpha fetoprotein 6.2 *µ*g/L, (beta HCG) beta human chorionic gonadotropin <3 IU/L, (LDH) lactase dehydrogenase levels 572 U/L, and serum testosterone 375 ng/dL, and all prognostic markers are within normal limits. CT of the chest, abdomen, and pelvis was negative for evidence of metastatic disease or lymphadenopathy.

**Figure 2:**
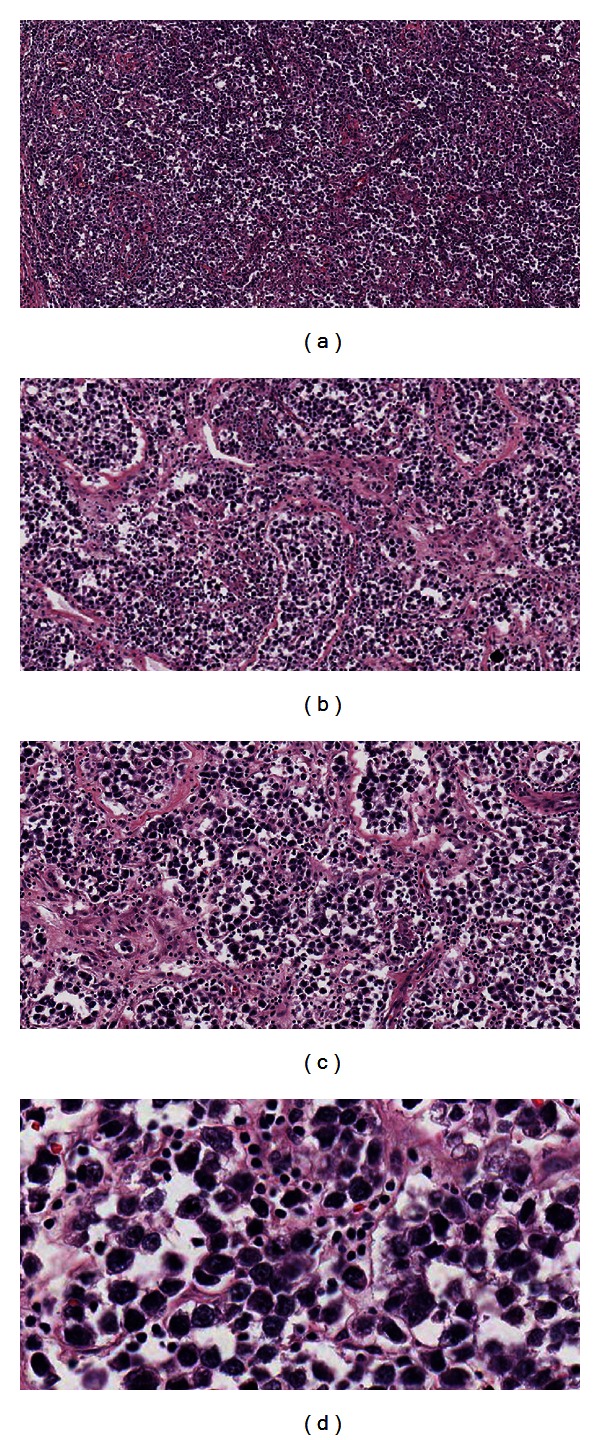
Surgical pathology. (a) Right testicle low power 4x, (b) left testicle low-power 4x, (c) 10x, and (d) 20x.

He underwent bilateral inguinal orchiectomy. Final pathology (Figure 2) revealed classical seminoma in both specimens. Both right and left tumors exhibited invasion of the rete testis. There was possible angiolymphatic space invasion noted within the left testicular mass. There was no tumor extension through the tunica albuginea, epididymis, or spermatic cord. Surgical margins were free. Both tumors were pathologically staged IA.

**Figure 3:**
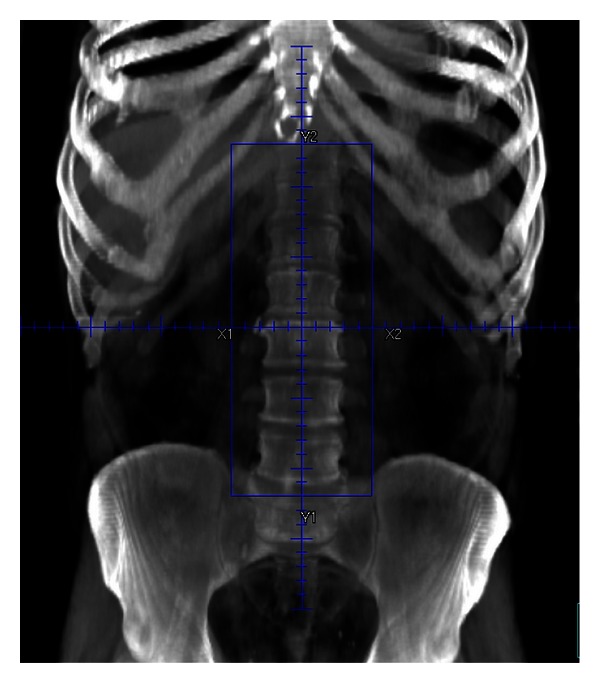
Paraaortic field (DCR).

The patient did not have concerns regarding fertility and declined to consider an organ preserving approach. Our patient was uncomfortable with surveillance as an option. He declined medical oncology referral for discussion about systemic therapy and was therefore treated with external beam radiotherapy. He received 2550 cGy in 17 fractions at 150 cGy per fraction via 6 MV/18 MV photons with AP/PA fields. Field borders (Figure 3) included superior border at *T*10/11 interspace, inferior border at L5/S1 interspace, and lateral borders of vertebral transverse processes (field width approximately 10 cm). The patient did not have a history of previous pelvic surgery, and only the paraaortic lymph nodes were targeted. He tolerated treatment well, experiencing grade I nausea (as per Common Terminology Criteria for Adverse Events, version 3.0). He is currently disease-free, 18 months from completion of radiation therapy. His serum testosterone levels fell to 248 ng/dL after treatment, and he uses testosterone gel for hormone replacement.

## 3. Discussion

Risk factors for development of testicular cancer have been reported by Dieckmann et al. and are described by a relative risk, odds ratio and include undescended testis (3.5–17.1,), contralateral GCT (24.8–27.6,), familial testis cancer (2.1–12.3,), and gonadal dysgenesis (up to 25% cumulative risk.) Other risk factors which have less evidence include dizygotic twin ship (1.5–2.4), infertility (1.6–10), and testicular atrophy (2.7–12.7) [13]. Incidence of testicular intratubular neoplasia (TIN) in the contralateral testes at the time of diagnosis of GCT has been reported at a rate of 6.6% [14]. Some believe that biopsy of the contralateral tumor at the time of orchiectomy is appropriate, while others do not recommend this as common practice [15]. Since the incidence of metachronous GCT is less than the incidence of TIN, one can conclude that TIN does not necessarily lead to cancer. BGCTs are reported to occur in a younger population compared to testicular cancer as a whole, and by one report, at a median age of 29 years versus median age of 34 years for solitary testicular GCT [14]. Although once debated in the literature, BGCTs are likely not increasing in incidence, and the apparent increased number of metachronous tumors is probably due to increased life expectancy of the general population [10]. BGCTs are reported consistently at a very low rate, previously mentioned, in Table 1, and although they occur usually within five years, they may occur much later, and therefore long term follow-up is recommended. In the largest U.S. series of BGCT, the author reports the diagnosis of the second lesion appearing at a time period greater than ten years after the original diagnosis in 23% of the cohort [10]. The incidence of metachronous tumors is two to ten times higher than synchronous tumors. Synchronous tumors are as likely to harbor divergent histology, as in Table 2. Seminoma is believed to be more commonly involved in the case of BGCTs as opposed to nonseminoma [16]. Synchronous tumors were once thought to represent more advanced staged disease, but this has not been demonstrated in the multiple series reviewed in this paper.

The synchronous presentation of bilateral stage I testicular seminomatous germ cell tumors presents a unique discussion regarding the role of and need for adjuvant therapy. Adjuvant radiation therapy after inguinal orchiectomy has resulted in excellent local control. After five years, recurrence, free survival and cause-specific survival are consistently reported at 95% and 98-99%, respectively [17, 18]. Doses reductions from 30 Gy to 20 Gy were achieved without compromising outcomes [19]. Furthermore, treatment to the paraaortic field was found to be equivalent to the dogleg, in a pelvis not previously disrupted with surgery [20]. Current radiation therapy standards include doses of 20–25 Gy at 1.5–2.0 Gy/day, treated with AP/PA fields, directed to the retroperitoneal lymph nodes, typically T10/T11 through L5 S1, 8–10 cm wide, and with consideration for accounting for left renal vein/IVC confluence (Figure 3). While radiotherapy has been excellent at preventing recurrence, chemotherapy with carboplatin is also very effective, possibly with a more favorable toxicity profile.

Platinum-based chemotherapy regiments are effective at preventing recurrence of seminoma and are generally well tolerated. In a randomized study, carboplatin (AUC 7 × 1 cycle) was found not to be inferior to radiotherapy with regards to 5-year RFS (94.7% versus 96.0%.) In addition, at a median followup of 6.5 years, the carboplatin arm experienced a reduced number of contralateral GCT compared to the radiotherapy arm, HR 0.22 (*P* = 0.03) [21]. Chemotherapy may confer an advantage to radiotherapy in that less patients experience a metachronous testicular tumor. Others have suggested that chemotherapy is not effective at preventing the incidence of a metachronous GCT [12]. For patients with synchronous germ cell tumors who undergo bilateral orchiectomy, at least short term, this advantage of chemotherapy no longer would exist.

If radiation therapy is used in the initial management of a patient, this would probably limit the ability to reirradiate in the event of onset of contralateral metachronous GCT years later, due to approaching the tolerance of small bowel. Regardless of how an initial GCT was managed, a population-based US study revealed that incidence of a second testicular GCT did not decrease survival [15]. Therefore, the increased incidence of metachronous GCT after radiotherapy compared systemic therapy should not be a major factor determining a treatment regimen.

Furthermore, surveillance, now accepted as category I evidence in the USA, is employed routinely after orchiectomy for stage I seminoma [22]. In a US cohort, managed with observation, at five years, men experienced 89.2% RFS, 98.8% OS, and 100% CSS [23]. Aparicio et al. described a risk-adapted approach, placing patient with stage I seminomas, <4 cm, without rete testis invasion on a surveillance protocol. They reported a 3-year DFS of 88.1%, all of which were recurred in the retroperitoneum and were salvaged with Etoposide and Cisplatin chemotherapy. Three-year overall survival was 100%  [24]. We are not aware of any literature that supports surveillance for this rare tumor.

Bilateral radical inguinal orchiectomy is considered the standard of care for patients with bilateral testicular germ cell tumors. Given the resultant infertility and need for indefinite androgen replacement therapy, some groups have proposed an organ preservation approach in selected patients. However, this remains controversial and goes against oncologic principles [25–28]. Potential candidates for organ preservation include those patients with organ confined bilateral tumors or tumors within a solitary testicle [27]. Tumors larger than 2 cm are rarely amenable to partial orchiectomy because total tumor excision often leaves insufficient remaining viable testicular parenchyma. Tomita et al. detailed a particular strategy for organ preservation in eight patients with bilateral testicular tumors. In these patients, radical inguinal orchiectomy was performed for the larger testis tumor. If pathology confirmed seminoma, then patients had their contralateral testis spared and received chemotherapy (three cycles of Bleomycin, Etoposide, and Cisplatin). Local control was maintained for the cohort, although one patient died of distant disease [29]. When utilizing testis preservation approaches, close and frequent postoperative surveillance with scrotal ultrasound has been suggested [25]. In general, testis-sparing strategies for testicular germ cell tumors are controversial but may be considered in highly selected patients. In our case, the patient declined preservation as an option.

Of the reported cases of bilateral stage I synchronous GCT, with concordant seminoma histology (Table 2), most of them were treated with bilateral orchiectomy and adjuvant radiotherapy. The Geczi series did not delineate patients with bilateral stage I, patients with concordant seminoma, but patients of the 19 synchronous BGCT; 5 were treated with adjuvant radiation [9]. Of the 2 cases of synchronous stage I seminomatous BGCT in the Hentrich series, one was treated with radiotherapy and the other with chemotherapy [12]. Otherwise, radiation therapy was used in all the others noted series for this particularly rare scenario. Dose, fractionation, and field size records were not generally reported [2–4, 6, 7, 9–12].

For bilateral stage I synchronous testicular germ cell tumors, the current standard of care is to perform bilateral radical inguinal orchiectomy, and then to consider adjuvant therapy. In the current case, adjuvant radiotherapy was recommended due to bilateral rete testis invasion and possible angiolymphatic space invasion of the larger lesion. Perhaps the presence of bilateral germ cell tumors is a negative prognostic factor, yet this may be difficult to demonstrate due to the small number of cases. Although morbidity associated with low-dose radiotherapy directed at the abdomen is not negligible, the adjuvant paraaortic radiotherapy remains safe and well-tolerated treatment regime.

## 4. Conclusions

For patients with stage I seminoma, surveillance, radiotherapy, or chemotherapy is reasonable options following orchiectomy. These patients have a good prognosis, regardless of treatment choice. Bilateral synchronous stage I seminoma of the testes is rare, with few cases reported in the literature. Our patient was treated with radiotherapy, like a majority of these patients have been managed historically. Bilateral orchiectomy is standard of care. Organ preservation remains investigational but may be considered for selected patients. Similar prognostic factors should be considered for adjuvant therapy for bilateral testicular germ cell tumors when compared to unilateral germ cell tumors. Surveillance has not been described in patients with bilateral germ cell tumors after orchiectomy. Chemotherapy is probably a reasonable option. Due to the lack of evidence, we propose that patients with bilateral stage I synchronous GCT, with concordant classical seminoma histology, should be managed with bilateral orchiectomy, followed by paraaortic radiotherapy.

## Figures and Tables

**Table 1:** Incidence of bilateral germ cell tumor in select series.

Author (reference)	Total number testicular germ cell tumors	Bilateral germ cell tumor (BGCT) (%)
Patel et al. [2]	795	19 (2.3)
Wanderås et al. [3]	2225	68 (3.0)
Coogan et al. [4]	2088	21 (1.0)
Sonneveld et al. [5]	445	16 (3.6)
Ondrus et al. [6]	960	27 (2.8)
Che et al. [7]	2431	24 (1.0)
Ohyama et al. [8]	274	9 (3.2)
Géczi et al. [9]	2386	72 (3.0)
Holzbeierlein et al. [10]	3984	58 (1.5)
Theodore et al. [11]	2383	45 (1.9)
Hentrich et al. [12]	1180	47 (4.0)

**Table 2:** Select series, incidence of BGCT and synchronous stage I seminomas, therapy, and outcomes.

Author	Years studied	BGCT	S-BGCT	CS-BGCT	CS-BGCT Stage I	Adjuvant radiation	FS	Dose (Gy)	Outcome
Patel et al. [2]	1935–44; 1977–86	19	4	2	2	2/2	—	—	***Died in <2 yrs
Wanderås et al. [3]	1953-1990	68	8	7	7/8 all stages	Likely All got RT	**Inver- ted Y	30–40	—
Coogan et al. [4]	Unknown	21	5	3	2	2/2	—	—	NED
Ondrus et al. [6]	1977–2001	27	3	1	1	1/1	—	—	NED
Che et al. [7]	1978–1999	24	4	3	3	3/3	—	—	NED
Géczi et al. [9]	1988–1998	72	19	13	*8	5/19	—	—	5 yr OS 84%
Holzbeierlein et al. [10]	1950–2001	58	10	3	2/3	2/2	—	—	—
Theodore et al. [11]	1997–2002	45	14	9	1	—	—	—	—
Hentrich et al. [12]	1979–2003	47	9	4	2	1/2	—	—	NED

BGCT: bilateral germ cell tumor; S-BGCT: synchronous bilateral germ cell tumor; CS-BGCT: classic seminoma, synchronous bilateral germ cell tumor; FS: field size; *did not differentiate those who were CS-BGCT and stage I; **pre 1980, included groins in anterior field for higher risk patients; ***both who died in <2 yrs were treated in 1935–1944 era; —: not reported.
